# Velocity spectrum imaging using radial k-t SPIRiT

**DOI:** 10.1186/1532-429X-14-S1-W59

**Published:** 2012-02-01

**Authors:** Claudio Santelli, Sebastian Kozerke, Tobias Schaeffter

**Affiliations:** 1 Division of Biomedical Engineering and Imaging Sciences, King's College London, London, UK; 2 Institute for Biomedical Engineering, University and ETH Zurich, Zurich, Switzerland

## Background

Fourier velocity encoding (FVE) [P.R.Moran,MRI(1),1982] assesses the distribution of velocities within a voxel by acquiring a range of velocity encodes (k_v_) points. The ability to measure intra-voxel phase dispersion, however, comes at the expense of clinically infeasible scan times. We have recently extended [C.Santelli,ESMRMB(345),2011] the auto-calibrating parallel imaging technique SPIRiT [M.Lustig,MRM(64),2010] to exploit temporal correlations in dynamic k-t signal space and successfully applied it to radially undersampled FVE data. Prior assumption of Gaussian velocity spectra additionally allows undersampling along the velocity encoding dimensions [P.Dyverfeldt,MRM(56),2006]. In this work, a scheme is proposed to non-uniformly undersample the k_v_-axes in addition to undersampling k-t space for reconstructing mean and standard deviation (SD) of the velocity spectra for each voxel in aortic flow measurements.

## Methods

### Acquisition

**Figure 1:**
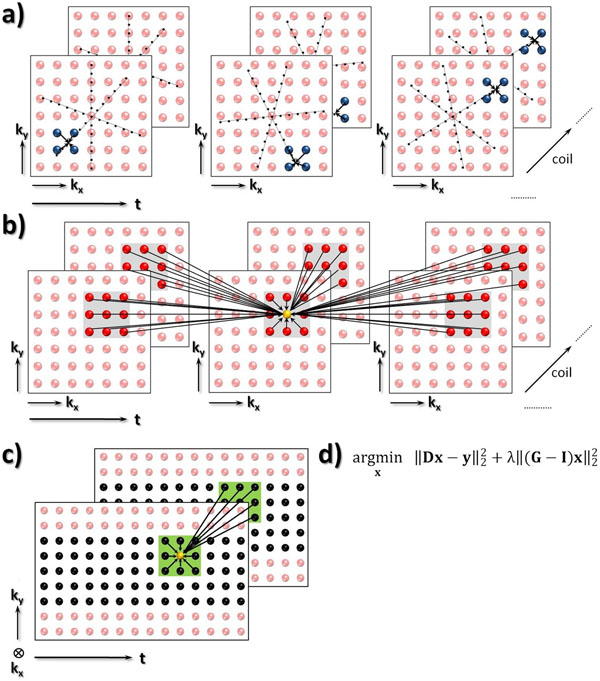
**a)** Dynamic Golden-angle acquisition providing an optimal distritbution of radial profiles [S.Winkelmann,IEEETransMedIm(26),2007]. **D** relates the reconstructed Cartesian k-t data, **x**, to the measured k-t points, **y**. **b)** Every Cartesian k-t sample point is expressed as linear combination of neighboring points in dynamic k-t space across all coils. **c)** The shift-invariant interpolation kernel weights (indicated by the arrows in the green neighborhood-mask) in **G** are obtained by fitting them to the fully sampled k-t calibration area in a Tikhonov-regularized least-squares sense. **d)** Unconstrained Lagrangian, where **I** denotes identity and λ a regularization parameter. The latter was set to 0.125.

2D radial (FOV=250mmx250mm) fully sampled cine FVE data of the aortic arch for 3 orthogonal velocity components was obtained from 5 healthy volunteers on a 3T Philips Achieva scanner (Philips Healthcare, Best, The Netherlands) using a 6 element receive array. Three different first gradient moments corresponding to encoding velocities of 25cm/s, 50cm/s and 200cm/s were applied along with a reference point (k_v_=0). Undersampled radial data sets were obtained by separately re-gridding these 4-point measurements onto Golden-angle profiles (Fig.1a).

### Reconstruction

The interpolation operator **G**, enforcing consistency between calibration data from a fully sampled centre of k-space and reconstructed Cartesian k-space points, **x**, is extended for dynamic MRI by including temporal correlations between adjacent data frames (Fig.1b). Data consistency is imposed using gridding-operator **D** (Fig.1a). Then, **x** is recovered by solving the minimization problem in Fig.1d). Reconstruction was performed for every k_v_-point separately using dedicated software implemented in Matlab (Natick,MA,USA). A 7x7x3 neighborhood in k_x_-k_y_-t space was chosen for the k-t space interpolation kernel. The weights were calculated from a 30x30x(nr cardiac phases) calibration area (Fig.1c). Mean and SD of velocity distributions were calculated for the resulting coil-combined images.

## Results

**Figure 2:**
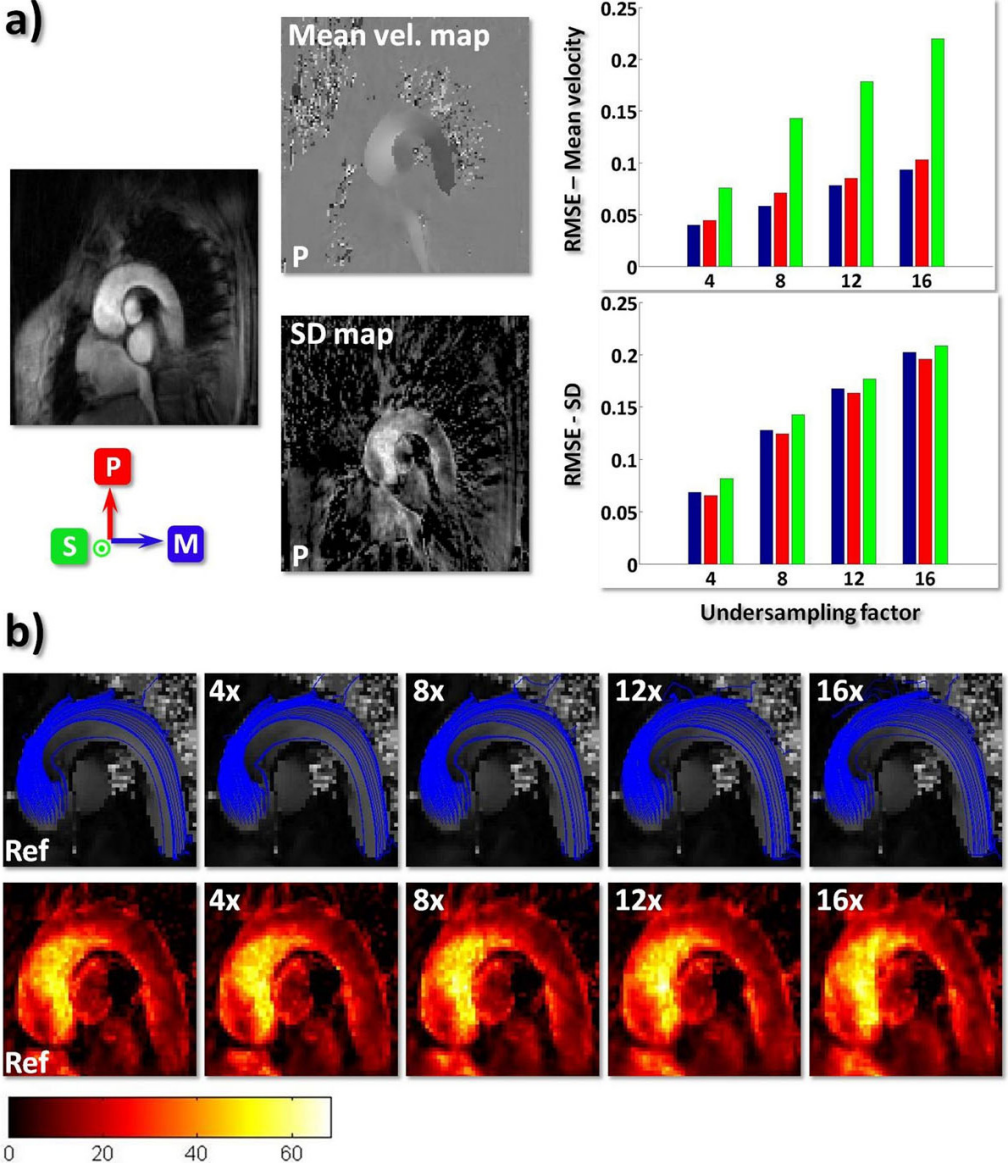
**a)** Coil-combined magnitude frame from fully sampled reference data with plots of mean velocities and SDs along phase encoding direction P. RMSE of reconstructed mean velocities (top) and SDs (bottom) from a ROI placed over the aortic arch and averaged over all time frames and volunteers. For each component, means were derived with a 3-point method [A.T.Lee, MRM(33), 1995] whereas SDs were fitted to all 4-point measurements in a least-squares sense. The mean velocity RMSE of the in-plane flow directions (M-P) is significantly smaller compared to the S-component. **b)** Top row: Stream lines derived from reference and undersampled systolic data plotted over velocity magnitude map. Bottom row: Corresponding turbulence intensity maps [J/m^3^] calculated according to [P.Dyverfeldt, JMRI(28), 2008]. The streamlines are congruent with the turbulence and phase dispersion maps, respectively.

Fig.2a) compares the mean root-mean-square error (RMSE) of the reconstructed mean velocities and SDs in the aortic arch for different undersampling factors and for each flow direction (M-P-S). Fig.2b) shows in-plane streamlines reconstructed from the acquired mean velocities and turbulence intensity maps calculated from SD values.

## Conclusions

A novel auto-calibrating reconstruction technique for dynamic radial imaging was successfully applied to undersampled 4-point FVE data from five healthy volunteers. Results show that up to 12-fold radial undersampling provides accurate quantification of mean velocities and turbulence intensities derived from velocity spectra.

